# Which of available selective serotonin reuptake inhibitors (SSRIs) is more effective in treatment of premature ejaculation? A randomized clinical trial

**DOI:** 10.1590/S1677-5538.IBJU.2019.0121

**Published:** 2019-12-17

**Authors:** Soheila Siroosbakht, Sadra Rezakhaniha, Bijan Rezakhaniha

**Affiliations:** 1 Faculty of Medicine, Imam Reza Hospital, AJA University of Medical Sciences, Tehran, Iran; 2 Department of Nutrition, Science and Research Islamic Azad University, Tehran, Iran; 3 Department of Urology, Imam Reza Hospital, AJA University of Medical Sciences, Tehran, Iran

**Keywords:** Citalopram, Fluoxetine, Premature Ejaculation

## Abstract

**Purpose:**

To compare the efficacy and safety of available selective serotonin reuptake inhibitors (SSRIs) in order to find the most effective drug with the least number of side effects in treatment of premature ejaculation (PE).

**Materials and Methods:**

This study was a randomized clinical trial. Four hundred and eighty patients with PE in the 4 groups referred to Imam Reza hospital Tehran, Iran from July 2018 to February 2019 were enrolled in the study. The patients received sertraline 50mg, fluoxetine 20mg, paroxetine 20mg and citalopram 20mg, every 12 hours daily. The intravaginal ejaculatory latency time (IELT) before treatment, fourth and eighth weeks after treatment was recorded by the patient's wife with a stopwatch.

**Results:**

Mean IELT before, 4 and 8 weeks after treatment in four groups were: sertraline 69.4±54.3, 353.5±190.4, 376.3±143.5; fluoxetine 75.5±64.3, 255.4±168.2, 314.8±190.4; paroxetine 71.5±69.1, 320.7±198.3, 379.9±154.3; citalopram 90.39±79.3, 279.9±192.1, 282.5±171.1 seconds, respectively. The ejaculation time significantly increased in all groups (p <0.05), but there was no significant difference between the groups (P=0.75). Also, there was no significant difference in drugs side effects between groups (p >0.05). The most common side effects were drowsiness and dyspepsia, which were not severe enough to cause discontinuation of the drug.

**Conclusions:**

All available SSRIs were effective and usually had no serious complications. In patients who did not respond to any of these drugs, other SSRI drugs could be used as a salvage therapy.

## INTRODUCTION

The most common type of sexual dysfunction in men is premature ejaculation (PE) (1). PE, such as delayed ejaculation is concerning to patients and their partners (2). No standard time is defined for ejaculation. Depending on the satisfaction of couples in sexual relationship, the ejaculation time varies from couple to couple (3). On the basis of the report of the second International Society for Sexual Medicine meeting (ISSM) for the definition of PE: (1) lifelong PE is defined as ejaculation that always or nearly always occurs prior to or within about 1 minute of vaginal penetration from the first sexual experience; and acquired PE is a clinically significant and bothersome decrement in latency time, often to about 3 minutes or less; (2) in all or nearly all vaginal penetrations, the patients have no ability to delay ejaculation; and (3), leading to distress, bother, disappointment, behavioral disorders or the avoidance of confidence sexual relationship and other negative personal consequences. Men with acquired PE are older, have cardiovascular disorders, comorbid diseases and erectile dysfunction (4, 5).

About one third of men suffers from premature ejaculation (6). In most of these people, there is no clear cause, but probably includes environmental and neurobiological factors (7). Psychological factors such as anxiety, feeling guilty or depression can also lead to premature ejaculation (8, 9). In some cases, premature ejaculation may occur as a result of medical factors including hormonal problems, physical injuries, or side effects of some drugs, especially antidepressant and antipsychotic compounds that were commonly underdiagnosed and underestimated by physicians (10). In these patients, the lack of close and intimate relationships between couples and the mental and physical health deterioration can be accompanied by either poor sexual life or a more frequent risky sexual behavior than other people in society. Shorter intravaginal ejaculatory latency time (IELT) causes more problems. Therefore, it is very important to diagnose and cure this problem (11, 12).

PE treatment was previously limited to behavioral therapy but due to knowledge about the role of serotonin in the central nervous system in control of ejaculation, has expanded to drug therapies over time (13, 14). Nowadays, there are several types of treatment for this problem, many of which are commercial drugs that are not confirmed by the World Health Organization and Ministry of Health (15). The drugs used for treatment of premature ejaculation are divided into two groups, including topical anesthetics and oral medications which are prescribed with regard to the patient's conditions (16). Today, gold standard treatment for PE is selective serotonin reuptake inhibitors (SSRIs) including citalopram, fluoxetine, sertraline, paroxetine and dapoxetine. Other drugs, such as tramadol and phosphodiesterase type 5 inhibitors (PDE5is) and antidepressants are also used. PDE5is is recommended in PE when accompanied with erectile dysfunction (17). Drugs are prescribed in two ways: daily intake and on-demand. The IELT is less increased with on-demand compared to daily intake, but may fulfill a suitable treatment for specific patients (18).

Fluoxetine is the leading SSRI due to its longer half-life. All drugs in this group require liver metabolism and have the half-life of 18 to 24 hours. But fluoxetine creates an active metabolite with the half-life of a few days. Other members of this group such as sertraline, citalopram, fluvoxamine and paroxetine do not produce long-effect metabolites. The most common side effects of these drugs are nausea, headache, anxiety, uneasiness, insomnia, and sexual dysfunction. The deprivation syndrome has also been described for SSRIs that might cause nausea, dizziness, anxiety, trembling, and heartbeat. The selective serotonin reuptake inhibitors are inhibitors of liver cytochrome P450 enzymes and this leads to the increase in metabolism of other drugs including tricyclic antidepressants and warfarin (19).

Due to the importance of PE and its effects on the individual and family relationship of the affected person, treatment of PE is imperative. Many types of SSRIs such as fluoxetine, sertraline, citalopram and paroxetine are widely used in the treatment of PE but there is, however, no consensus and agreement on the type, dose, duration of treatment and side effects. Different results have been obtained from previous studies, and this shows the importance of further investigations to achieve more definite and conclusive results. Therefore, we decided to evaluate, outline and compare the efficacy and safety of currently available SSRIs in order to find the most effective and least complicated drug. Most previous studies compared one or two drugs but in this study, four common and available drugs were evaluated.

## MATERIALS AND METHODS

### Study populations

This study was a randomized clinical trial without placebo drug group. The study population included 480 patients with PE referred to Imam Reza Hospital Tehran, Iran from July 2018 to February 2019. The sampling method was convenience. Patients were evaluated by an urologist and, if they had premature ejaculation according to the definition of ISSM, they would be enrolled in the study. Randomization method in this research was simple randomization. Randomization unit was individual and patient's randomization was done by computerized random numbers. The starting point was completely random (selecting a number on the table with closed eyes) and the direction of movement in the table was selected to the bottom. The patients were randomly assigned to one of the four study groups (each group of one hundred twenty) using the random numbers table and received the relevant intervention. Inclusion criteria were male with premature ejaculation at least one coitus per week and age between 20 to 75 years. Exclusion criteria were neurologic disorder psychological disorder age less than 20 and over 75 years urinary and genital infection and history of pelvic surgery, diabetes, alcohol and drug abuse, erectile dysfunction and the use of PDE5is.

### Intervention groups

First group received 50mg sertraline, second group 20mg fluoxetine, third group 20mg paroxetine and forth group 20mg citalopram, every 12 hours daily (Sobhan Daro Company, Rasht, Iran). Finally, the time to ejaculation before treatment (mean time in at least three coitus) and at the end of the fourth and eighth weeks after treatment were accurately measured and recorded by the patient's wife with a stopwatch. Patients were advised to record time from vaginal entrance to ejaculation.

### Main outcome measures

Main outcome measure used in this study was intravaginal ejaculatory latency time (IELT) and efficacy and side effects of currently available drugs for PE.

### Statistical analysis

Data analyses were performed by SPSS statistical software version 24. For qualitative variables, frequency and frequency percent and for quantitative variables mean and standard deviations were calculated. The paired sample t-test was used for evaluation of IELT before, 4 and 8 weeks after treatment in each group and the Pearson correlation coefficient test was used for the evaluation of the IELT between four groups. Chi-square test was used to evaluate the side effects. P value <0.05 was considered significant.

## RESULTS

In this study, 480 patients with mean age of 36.5 years with SD of 11.41 were studied. The mean age of the patients were matched in four groups (Table-1). Forty-six percent of patients had academic education and the mean number of coitus per week in four groups was 1.84. All patients were matched for education level and coitus number 82.5% of sertraline group, 71.7% in fluoxetine group, 81.7% in paroxetine group and 81.65% in citalopram group were married. Mean IELT before, 4 and 8 weeks after treatment in four groups were: in sertraline group 69.4±54.3, 353.5±190.4 and 376.3±143.5 second; in fluoxetine group 75.5±64.3, 255.4±168.2 and 314.8±190.4 second; in paroxetine group 71.5±69.1, 320.7±198.3 and 379.9±154.3 second, in citalopram group 90.39±79.3, 279.9±192.1 and 282.5±171.1 second, respectively. Overall, the frequency of response to treatment in groups was as follow: sertraline 92.5%, fluoxetine 84.2%, paroxetine 93.3% and citalopram 91.8%. There was no significant difference between the four groups in the time of ejaculation before treatment (p=0.32). As it was seen, the ejaculation time significantly increased in all four groups (p <0.05), but there was no significant difference between the four groups (P=0.75) (Table-2). Frequency of side effects in four groups was: sertraline 15%, fluoxetine 20.8%, paroxetine 12.5% and citalopram 10%. Although there was no significant difference in drugs side effects between groups (p >0.05), the percent of side effects in fluoxetine group was higher than other groups (Table-3). It should be noted that none of the side effects of the drugs were severe enough to cause it to be discontinued.

**Table 1:** Mean age of groups*.

Group	Number	Minimum	Maximum	Mean	Standard Deviation
Sertraline	120	20	64	38.5	12
Fluoxetine	120	20	75	35.2	11.2
Paroxetine	120	22	69	37.5	10.9
Citalopram	120	21	74	34	11.6

* The patients of four groups were matched by age. No significant difference was observed between the four groups (p=0.73).

**Table 2:** Mean and standard deviation (Second) of IELT* in four groups†.

Group	Sertraline	Fluoxetine	Paroxetine	Citalopram	P Value
Before	69.4±54.3	75.5±64.3	71.5±69.1	90.39±79.3	0.32
4 week later	353.5±190.4‡	255.4±168.2‡	320.7±198.3‡	279.9±192.1‡	0.75
8 week later	376.3±143.5§	314.8±190.4¶	379.9±154.3§	282.5±171.1§	0.75

* Intravaginal Ejaculatory Latency Time

† The IELT significantly increased in all four groups (p <0.05), but there was no significant difference between the four groups (P=0.75)

‡ Significant difference with P value 0.0001

§ Significant difference with P value 0.006

¶ Significant difference with P value 0.000

**Table 3:** Side effects in four groups*.

Frequency Side effect	Sertraline 15%	Fluoxetine 20.8%	Paroxetine 12.5%	Citalopram 10%	P Values 0.05†
Insomnia	2	2	2	1	
Drowsiness	3	6	3	4	
Dyspepsia	3	2	2	3	
Constipation	2	_	1	^_^	
Nausea	2	2	2	1	
Loss of Appetite	1	2	1	1	
Fatigue	2	2	1	^_^	
Headache	1	2	1	1	
Vertigo	1	1	-	1	
Anxiety	1	2	-	-	
Urinary Retention	-	2	1	-	
Loss of Sexual Desire	-	2	1	-	

* Number of Patients

† There was no significant difference in drugs side effects between groups (p >0.05), but the percent of side effects in fluoxetine group was higher than other groups.

## DISCUSSION

Lifelong and acquired PE is the most common type of sexual dysfunction in men. Many types of SSRIs such as fluoxetine, sertraline, citalopram and paroxetine are widely used in the treatment of PE (17–19). Different results have been obtained from previous studies. Therefore, we compared the efficacy and safety of currently available SSRIs twice daily to find the most effective drug with the least number of side effects. In the most previous study, only one or two drugs were compared but in this study four common and available drugs were studied in Iran (16, 20–24). In the current study, the response rate to treatment in groups was as follow: sertraline 92.5%, fluoxetine 84.2%, paroxetine 93.3% and citalopram 91.8%. The IELT significantly increased in all four groups (p <0.05), but there was no significant difference between the four groups (P=0.75).

Another finding of this study showed that the side effects in four groups were not significantly different. The percentage of drug side effect in four groups were: sertraline 15%, fluoxetine 20.8%, paroxetine 12.5% and citalopram 10%. Although there was no significant difference in drugs side effects between groups (p >0.05), the percent of side effects in fluoxetine group was higher than other groups. The cause of the higher incidence of side effects of fluoxetine may be due to its active metabolite with the half-life of a few days, which is not seen in others. Sertralin, citalopram, and paroxetine do not produce long-effect metabolites (19). Also, this study showed that none of the side effects of the drugs were severe enough to cause it to be discontinued.

Rezakhaniha studied the effect of citalopram in PE (63 patients received citalopram 40mg in daily and 53 patients, 20mg on-demand). This study showed that both methods were effective but daily use was more effective (20). The side effects were 26% in daily and 20% in on-demand. In our study, 480 patients were evaluated in 4 groups of drugs and the rate of side effects in citalopram group were 10%. Turgay et al. compared the sertraline (50mg) and citalopram (20mg) daily in 80 patients with PE. He showed that both drugs were effective and safe. In our study, citalopram 40mg/day and sertraline 100mg/day were used. By comparing the IELT after treatment, there was no significant difference between the two studies, which indicated that less dose of drugs was also effective in the treatment (21).

In another study, 43 patients received fluoxetine and 34 patients received citalopram daily for 4 weeks, both of which were effective in increasing the IELT. In this study, adverse effects were not studied (22). However, in our study, 480 patients in four groups of drugs were studied for 8 weeks, and rate of side effect was also assessed in each group separately. In clinical practice, follow-up side effects are an important part of the evaluation of treatment for PE (25). Dadfar et al. reported that citalopram can be used in patients who did not respond to fluoxetine as a salvage drug (23). According to our results, patients who do not respond well to a drug can use other drugs of SSRI. Khosh-del et al. in a study on 49 patients, confirmed that fluoxetine was effective in increasing the IELT in both methods, daily and on-demand. The complication of daily intake was 44.9% and in on-demand 12.8%. But in our research, adverse effects of fluoxetine were 20.8% (24).

Wang et al. reported different results in their study, the efficacy of available SSRIs was uncertain and there was no consensus on the type and dose of these drugs (26). Jian et al. showed that topical delayed creams with PDE5is and SSRIs resulted in the highest IELT (27). Recently, in Europe, the delayed spray [lidocaine--prilocaine (Fortacin^®^)] is considered a real first line treatment for PE because of its unique preparation, making customer friendly and its easy-handling (28). The acupuncture has shown benefits in limited studies in the treatment of PE and additional studies are required to assess it (29).

Although other drugs, especially dapoxetine, have been introduced as drug of choice for treatment of PE that has short halflife (1.5 hour) and low bioavailability (42%), in clinical practice, drugs are chosen based on the availability of that drug in the country. In Iran, the most available drugs in treatment of PE are sertraline, fluoxetine, citalopram and paroxetine. Although dapoxetine is also found in Iran, but due to its cost issue and limited distribution, the currently available drugs such as citalopram, fluoxetine, sertraline and paroxetine are used as first-line drugs.

The limitation of our study was short duration of follow-up. If we increased the followup period to 6-12 months, the efficacy and side effects of the drugs would be better evaluated. Therefore, we suggest that further studies must be carried out with a longer follow-up period.

## CONCLUSIONS

According to the finding of this study, all of these available SSRIs are effective and usually have no serious complications. In patients who did not respond to any of these drugs, other drugs of SSRI could be used as a salvage therapy. Another finding of our research confirmed that severe adverse effects that cause discontinuation of drug were not observed. The most common side effects were drowsiness and dyspepsia. Therefore, if the patient had sleep disturbance or a history of gastrointestinal upset, they should use them with caution and if intolerable to the patient, an alternative drug should be used.

### Ethical considerations

In all stages of the study, ethical issues of observation, the name and information of patients were kept confidential. The ethics committee of Army University of Medical Sciences approved the research project of this study (Reg No: IR.AJAUMS.REC.1396.112).

### Clinical trial registrations

This study approved in Iranian Registry of Clinical Trials (IRCT id: IRCT20180401039167N2).

## ABBREVIATIONS

PE: premature ejaculation
IELT: intravaginal ejaculatory latency time
mg: milligram
ISSM: international society for sexual medicine
SSRIs: selective serotonin reuptake inhibitors
PDE5is: phosphodiesterase type 5 inhibitors
